# The multiple fates of gene duplications: Deletion, hypofunctionalization, subfunctionalization, neofunctionalization, dosage balance constraints, and neutral variation

**DOI:** 10.1093/plcell/koac076

**Published:** 2022-03-07

**Authors:** James A Birchler, Hua Yang

**Affiliations:** Division of Biological Sciences, University of Missouri, Columbia, Missouri 65211, USA; Division of Biological Sciences, University of Missouri, Columbia, Missouri 65211, USA

## Abstract

Gene duplications have long been recognized as a contributor to the evolution of genes with new functions. Multiple copies of genes can result from tandem duplication, from transposition to new chromosomes, or from whole-genome duplication (polyploidy). The most common fate is that one member of the pair is deleted to return the gene to the singleton state. Other paths involve the reduced expression of both copies (hypofunctionalization) that are held in duplicate to maintain sufficient quantity of function. The two copies can split functions (subfunctionalization) or can diverge to generate a new function (neofunctionalization). Retention of duplicates resulting from doubling of the whole genome occurs for genes involved with multicomponent interactions such as transcription factors and signal transduction components. In contrast, these classes of genes are underrepresented in small segmental duplications. This complementary pattern suggests that the balance of interactors affects the fate of the duplicate pair. We discuss the different mechanisms that maintain duplicated genes, which may change over time and intersect.

## Teaser

The fates of duplicate genes are discussed.

## Introduction

Mutations give rise to new variation and gene duplications give rise to new genes. New genes evolve from old genes. New genes foster the complexity of life leading to a multitude of new forms and functions. The trajectory of duplicate genes is usually a return to the singleton state, but retention can occur by various mechanisms. While there have been claims of completely de novo genes, they often include pre-existing domains that are rearranged. Rearranged domains from different proteins, of course, can contribute to new functions. A truly de novo gene that is not selected against might be one that is antagonistic to a normal function that somehow confers an advantage to the organism. Some of the best candidates might be microRNAs due to their small size and modulatory action (Lu, 2019). Nevertheless, duplication is the predominant means of generating new gene functions. Here we discuss the various fates of duplicated genes.

## Mechanisms of gene duplication (and deletion) 

Gene duplications can occur in tandem via recombination between displaced repetitive sequences on homologues, via transposition by aberrant transposable element mobilizations (Zhang et al., 2013; Wang et al., 2015), or by being carried by Mutator-like elements (Jiang et al., 2004) or helitrons (Lal et al., 2003; Lai et al., 2005; Jameson et al., 2008; Barbbaglia et al., 2012), although the products are often gene fragments. Complete duplicates are in the minority but have the most consequences caused by the altered dosage (Zhang et al., 2021). Once a tandem duplication occurs, it can expand to a triplication or be reduced back to the singleton state by asymmetric recombination between the two members. Alternatively, duplicates can result from whole-genome duplication (WGD) (Wolfe and Shields, 1997; Simillion et al., 2002; Bowers et al., 2003; Blanc and Wolfe, 2004; Maere et al., 2005; Freeling, 2009; Jiao et al., 2011). In the plant kingdom, WGDs have occurred many times in various evolutionary lineages. Essentially, all angiosperms have a history of multiple WGDs of various ages (Jiao et al., 2011). Thus, whether a plant species is to be considered a polyploid or diploid depends on when one starts counting, blurring this distinction on the molecular level. The fate of most gene duplications is that one member of the pair is deleted or loses function, but some forces work to maintain the duplicate state.

In order for genes to be maintained in a genome, they must provide a function to the organism. Numerous types of mutational processes such as deletion via illegitimate recombination, ultraviolet light, replication errors, or transposable element insertion occur continuously, resulting in deletion or deterioration of genes unless purifying selection acts against the loss of the encoded function. For example, fish restricted to caves have become eyeless, losing the function of this otherwise critical organ when it is of little use (Yamamoto, 2004). Similarly, plants that have gained the ability to obtain a carbon source from other species often lose their chlorophyll (Wolfe et al., 1992). Thus, perhaps the most common fate of duplicated genes is that one member is deleted to return the gene copy status to singleton (Woodhouse et al., 2010).

Gene function is maintained through natural selection. If we consider the notion that duplicate genes are maintained so that a “back up” copy is present in the genome or that duplicates “buffer” each other, this would require that one copy of the duplicate pair would have to regularly fail to perform its function without being deleted, so that the “back up” copy could be selected upon. Early after a duplication event, a nullifying mutation in one copy of nondosage-sensitive genes would likely be of no effect and could persist with the single functional copy still present. Mutational assault shortly after WGD could be withstood better by the newly minted polyploid than the diploid progenitors (Innan and Kondrashov, 2010) because multiple copies of a gene are present, but this fact is unlikely to be a force for maintaining the duplicate state and will no longer occur as the polyploid genome fractionates back to a near diploid.

Another possible circumstance is that homoeologous copies of genes in an allopolyploid can become identical. One possible mechanism for this occurrence might be gene conversion, for which an example has been documented in yeast (Evangelisti and Conant, 2010). However, this is difficult to distinguish from homoeologous shuffling with nullisomic/tetrasomic compensation that will homogenize gene copies and can be clearly documented on the chromosomal level (Xiong et al., 2011). Homoeologous shuffling would homogenize segments of chromosomes in a syntenic relationship, which would allow a distinction.

To some degree, the fate of duplicate genes depends on the nature of the mutations that occur in the two copies that provide grist for selection. Muller (1932) coined terms for various types of mutations that have influenced the terminology in considering gene duplication changes from the progenitor (Table 1). The categories with regard to the fate of duplications are not always clear and indeed might change over the life of the duplication. Thus, there is a need for definition of terms, but the ultimate interest is the forces at play that affect the flux of the gene repertoire over time and how this influences the evolution of the genome.

**Table 1:** Types of mutations in gene duplicates (defined by Muller, 1932)

Mutation type	Phenotype
Amorphic	Complete loss of function
Hypomorphic	Partial loss of function
Hypermorphic	Over-expression
Antimorphic	Antagonistic relative to wild-type
Neomorphic	New function

### Hypofunctionalization

**Figure 1:**
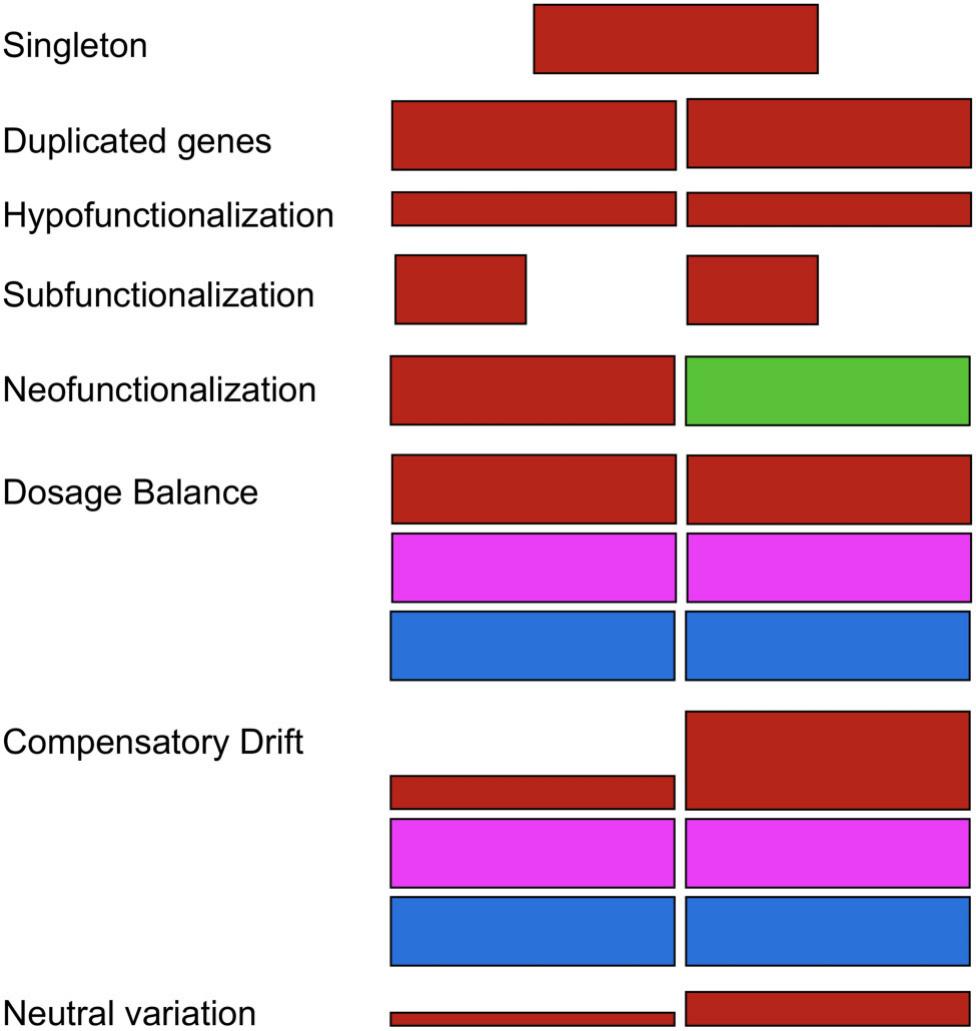
The fates of gene duplications. Starting from a singleton state a gene can become duplicated being then present in two copies via tandem duplication, transposed duplication, or WGD. Hypofunctionalization involves the reduction in expression of both copies of a duplicate pair to a threshold level at which both copies are needed for the specific function of the gene and thus both copies are maintained. Subfunctionalization is the division of function such that the two members of a pair confer only part of the functions of the progenitor singleton. Neofunctionalization is the diversification of one member of a duplicate pair for a new function. Dosage balance can mediate retention of specific classes of genes that are dosage sensitive to maintain their stoichiometric relationship with partners in multicomponent interactions. Nevertheless, compensatory drift refers to the observation that duplicates can diverge but depending on the total expression, a dosage balance effect will still operate with their partners. Neutral variation refers to the possibility that different members of a duplicate pair might have different expression levels under some circumstances in which the dosage or function is not an object of selection, similarly to apparent neutral variation of singleton genes.

A common trajectory of duplicated genes is the process of hypofunctionalization or dosage sharing (Qian et al., 2010; Gout and Lynch, 2015; Lan and Pritchard, 2016; Panchy et al., 2016; Veitia, 2017). In this case both members of a duplicate pair experience changes in expression level that reduce the total output (Figure 1). If the total amount of gene product is lowered to a level that is barely sufficient to supply the encoded function, then the duplicate pair would be maintained because the loss of either one of the two members of the pair would drop the total amount of gene expression below the threshold needed. The level of expression of the two members does not necessarily have to decline in parallel. Given that a certain level of gene expression for vital function differs across tissues, the members of the pair could diverge considerably in tissue-specific expression with only the critical tissues serving to maintain the level from both members to hold them in duplicate. Thus, the different expression levels of the duplicate pair are not necessarily a reflection of the partitioning of function when the hypofunctionalization is specific to a critical tissue. Another means by which hypofunctionalization might occur is if one member of a duplicate pair in a path to deterioration becomes antagonistic to the remaining copy (antimorph) and causes a partially reduced total amount of function (Blackman et al., 2010; Panchy et al., 2016) near to the required threshold level but not below. This circumstance, however, would not necessarily result in the maintenance of both members of the duplication if the antimorphic mutation is deleted. Hypofunctionalization could be reversed if additional mutations increase the expression level of one member of the pair for sufficient function at the critical life cycle stage followed by deletion of the other copy. Hypofunctionalization could be a path to subfunctionalization or neofunctionalization, described below, depending on the outcome of additional mutations.

### Neofunctionalization

The classical case of evolution by gene duplication involves circumstances in which duplicate genes diverge in their functions to provide new ones, an idea championed by Ohno (1970) (Figure 1). This process is referred to as neofunctionalization. When a new function arises that provides a successful evolutionary path, the duplicate pair of genes become fixed in the lineage. In this case, the pair of genes becomes maintained in duplicate essentially in perpetuity because the loss of either copy would be detrimental to the organism (Lynch and Conery, 2000).

The Drosophila bithorax complex is a classical example of neofunctionalization of duplicated genes, in which a set of homeobox genes is arranged in tandem to specify the identity of different body parts to the segments in which they are expressed (Lewis, 1951). Lewis (1951) postulated that these genes arose by tandem duplication and then diverged in function so that each now specifies the different identities of the segmented body plan. This critical innovation arose early in animal development and is the foundation for the body plan of segmented animals. Humans, for example, have four different clusters of these tandemly arrayed genes, which are thought to have arisen by two WGD events in the evolutionary lineage. An early described example in plants involves the diverged tissue-specific functions of a duplicated anthocyanin gene in maize (Stadler, 1946; Stadler and Emmerling, 1956).

Genes affecting floral morphology and secondary metabolism provide excellent examples of neofunctionalization in plants (Chaanderbali et al., 2016). Different related transcription factors control various aspects of flower development and timing in evolutionary lineages (Sharma and Kramer, 2012; Madrid et al., 2021). The genes encoding cytochrome P450 genes are particularly prolific in plant species with diverse functions involved with hormones, lignin synthesis, secondary metabolites, defense compounds, and other biochemical functions. There are 256 members of this gene family in the Arabidopsis genome (Cannon et al., 2004). Some of these are organized as tandem arrays with individual members of the array presumably catalyzing different reactions. Genes involved with defense mechanisms are particularly prone to divergence of duplicate copies.

Freeling et al. (2015) posed the important question of what qualifies as a “new” function for a member of a duplicate pair of genes. Indeed, there is a tendency in the literature to assign neofunctionalization to differences from hypermorphic, antimorphic, or neomorphic mutations as defined by Muller (1932), but it is in fact difficult to determine if these cases are the basis of “new” genes or neutral variation at the evolutionary timeframe of examination. Muller suggested the definition of a neomorphic mutation as one for which an additional normal copy would have no impact on the phenotype. While this might be a good guide for defining neofunctionalization, genes that are dosage sensitive such as transcription factors and signaling components complicate even this definition.

### Subfunctionalization

Subfunctionalization is related to neofunctionalization, but in this case, the duplicate pair partitions the functions of the progenitor gene (Figure 1). This process plays off the concept that genes typically will deteriorate and if there are two copies present, they may do so in different ways (Lynch and Conery, 2000). Thus, in order for all of the progenitor functions to be present, the duplicate pair must be retained in duplicate.

A classic example of subfunctionalization involves the hemoglobin cluster in humans (Hardison 2012). This oxygen-carrying protein has been duplicated by various means in vertebrate evolution. Some of the duplicated versions have taken on a different developmental timing but perform a similar function at the different stages. The copies with diverged functions are both required for normal function in the organism.

There is a tendency in the literature to ascribe subfunctionalization to any case in which duplicate genes have somewhat different expression patterns. Whether or not there has actually been a partitioning of function between the duplicate pair or whether this occurrence simply reflects the neutral variation of little to no consequence or hypofunctionalization is extremely difficult to assess (Panchy et al., 2016). Genes present in only a single copy in the genome can have allelic variation for the quantity or tissue-specific expression, which is likely evolutionarily neutral for the most part. Thus, when variation occurs for the quantity or tissue specificity of different members of a duplicate pair, this should not necessarily be ascribed to a partitioning of function. For duplicate genes of recent origin, it is particularly difficult to determine whether differences in expression reflect true subfunctionalization. The observation that most duplicate genes eventually transition back to a singleton state suggests that differences are not usually subfunctionalization. Duplicate genes that have persisted for millions of years with a maintenance of partitioned tissue specificity or other divided functions are candidates for subfunctionalization.

### Dosage balance

Another force that acts to maintain duplicate genes is dosage balance (Birchler et al., 2005; Birchler and Veitia 2012, 2021). When whole-genome sequences became available, it was apparent that certain classes of genes tend to be found in duplicate more often than others (Blanc and Wolfe, 2004; Freeling and Thomas, 2006; Li et al., 2016; Kuzmin et al., 2020). In general, these tend to be genes involved in multicomponent interactions that are sensitive to gene dosage. Typical examples include genes that encode transcription factors, signal transduction components, or chromatin proteins (Birchler et al., 2001). Subsequently, microRNA genes were also found to be generally retained (Zhang et al., 2009; Abrouk et al., 2012; Zhao et al., 2015; Peterson et al., 2021), which is consistent with their frequent function in modulating transcription factor expression.

Dosage-sensitive genes are deemed to be responsible for the effects of aneuploidy given that they mimic the impact of aneuploidy on the phenotype of eukaryotic organisms (Birchler et al., 2001; 2005; Birchler and Veitia, 2021). Aneuploidy, or the change in dosage of individual chromosomes or chromosomal segments, is detrimental with both increases and decreases of chromatin. Changing the entire set of chromosomes has less of an effect, which has been recognized for a century (Blakeslee, 1921). The impact of aneuploidy on global gene expression is typically an inverse or proportional modulation across the genome relative to the varied chromosomal region (Birchler, 1979; Birchler and Newton, 1981; Guo and Birchler, 1994; Sun et al., 2013a, 2013b; Hou et al., 2018; Birchler and Veitia, 2021). The same general relationship occurs for individual dosage-sensitive genes that are varied to the same copy number (Birchler et al., 2001; Birchler and Veitia, 2021). In contrast, less genome-wide modulation occurs from ploidy changes (Guo et al., 1996; Hou et al., 2018; Shi et al., 2021; Yang et al., 2021).

The preferential retention of dosage-sensitive genes was therefore postulated to be the result of negative fitness consequences if a member of an interacting group was deleted against the constant copy number of the interactors (Birchler et al., 2005; Freeling and Thomas, 2006; Veitia et al., 2008; 2013) (Figure 1). In other words, deletion of one member of a multicomponent interacting complex would be similar to an aneuploidy effect with similar detrimental consequences.

In a study of several plant species, Defoort et al. (2019) found that genes retained from WGD had a constraint for their level of expression. Genes retained from WGD showed an overrepresentation of involvement in protein–protein interactions whereas genes duplicated segmentally showed an underrepresentation of such interactions. In a study in Lotus with a single WGD (Shi et al., 2020), genes retained were the typical transcription factors and signaling components. These duplicates tended to have similar co-expression and a greater sequence conservation than segmental duplications. These studies support the concept that retention of selected classes of genes following WGD involves those associated with multicomponent interactions and that selection operates on variation that could upset the stoichiometry or balance of the components. Paralogs retained from WGD in rice and sorghum show fewer single-nucleotide polymorphisms than occur with tandem duplicates (Guo et al., 2019), illustrating the constraint on the former.

Certain classes of genes tend to return to single copy status through multiple polyploidy events (De Smet et al., 2013). This observation has been interpreted to indicate that these classes of genes have a tendency toward detrimental dominant mutations that would remove them if they have negative effects with regard to stoichiometric interactions in the cell.

#### Small-scale duplications

Analyses of classes of genes found in populations as segmental duplications reveal that those involved with multicomponent interactions are underrepresented, revealing a complementary pattern to those retained following WGD (Hakes et al., 2007; Freeling et al., 2008; Wang et al., 2011; Rodgers-Melnick et al., 2012; McGrath et al., 2014; Tasdighian et al., 2017). In this case, the increase of dosage-sensitive genes upon duplication would again mimic an aneuploid effect, be detrimental, and thus be selected against. The larger the size of the segmental duplication, the more likely it is to be detrimental. The most common effect of hyperploidy is an inverse modulation of global gene expression causing a reduced expression of a subset of genes across the genome. Larger segments of the genome that are varied in dosage tend to show a greater number of global modulations (Shi et al., 2021; Yang et al., 2021). This observation provides a molecular explanation for the detrimental effect of segmental duplication of dosage-sensitive genes.

Of course, if a small segmental duplication provides the organism with an exceptionally strong selective advantage, both copies might be retained, for example, if they confer disease resistance. Also, in some cases, absolute dosage can provide an advantageous increase of flux through a metabolic pathway that can be selected (Bekaert et al., 2011). The ultimate fate of a small segmental duplication will depend on the types and strength of selection operating on the increased copy number.

The complementary pattern of retained genes following WGD and segmental duplication is best explained by gene balance involving the stoichiometry of the interacting components of multicomponent cellular interactions, which if upset cause negative fitness consequences (Veitia 2002, 2003, 2004; Papp et al., 2003; Schuster-Bockler et al., 2010; Makanae et al., 2013; Robinson et al., 2021). Other potential explanations do not predict a complementary pattern of gene functions. Experimentally, the subtraction or addition of segments of the genome is more detrimental than a change in the whole genome (Hou et al., 2018; Shi et al., 2021; Yang et al., 2021), so the observed pattern of retained genes from WGD and segmental duplications provides a satisfying convergence between the evolutionary fate and experimental results.

## Genome dominance

When WGDs are of allopolyploid origin, a typical result is that there is a difference in the overall expression level of the two genomes in the new configuration (Wang et al., 2006; Flagel et al., 2008; Garsmeur et al., 2014). This difference can persist long after formation of the polyploid (Murat et al., 2014; Woodhouse et al., 2014; Renny-Byfield et al., 2015; Cheng et al., 2016; Edger et al., 2017). Despite this difference, the retention via dosage balance appears to operate (Thomas et al., 2006; Woodhouse et al., 2010). However, as the retention via dosage balance deteriorates over time, members of the duplicate pair from the lesser expressed genome tend to be those that become deleted (Thomas et al., 2006; Schnable et al., 2011, 2012; Cheng et al., 2012), leaving the more highly expressed member as the remaining singleton. In a comparative study between maize and soybean, the degree of genome dominance was greater in maize than soybean. This difference was suggested to be due to the subgenomes being more distinct in the progenitor species to maize compared to soybean (Zhao et al., 2017). A greater number of homoeologs are retained in soybean than in maize. In soybean, genes in pericentromeric regions have higher levels of expression than their homoeologs in chromosome arms (Du et al., 2012).

## Compensatory drift

While gene balance appears to be responsible for a large fraction of retained genes following WGD, the balance constraints can be fluid. This conclusion is supported by the observation that longer timespans from WGDs to the present are associated with fewer remaining gene duplicate pairs (Maere et al., 2005; Aury et al., 2006). New effective stoichiometries or balance among interacting components must evolve to account for this observation (Birchler et al., 2005). Indeed, a recent study (Shi et al., 2020) shows that the degree of connectivity of retained genes corresponds to the degree of sequence divergence illustrating how stoichiometric constraints can evolve to new relationships. The more interactors a gene has, the less sequence divergence occurs between the duplicate members. Fewer interactors leads to less selection pressure on the pair of genes. The latter case has a greater potential to evolve new functional stoichiometries with its interactors because as the strength of the constraint decreases, the opportunity for new effective balances to emerge increases.

While dosage balance operates to maintain gene duplicates following WGD, this observation does not necessarily mean that the duplicate members retain similar expression levels. Modeling illustrates that the levels of expression of individual members of a duplicate pair can change without upsetting the overall balance (Thompson et al., 2016; Figure 1). An example of this situation is illustrated by the expression of the paralogs of the *FLC* flowering genes in polyploid Brassica (Calderwood et al., 2021). In this case, the individual genes have divergent expression levels, but the total amount is maintained. As noted above, variation in expression levels of individual members of a duplicate pair might be neutral in some tissues, while having a critical level in specific cell types. In plants, the haploid gametophyte generation is likely to be a situation in which the stoichiometric effects are more critical than in the diploid sporophyte, given that aneuploidy in haploids has stronger effects than in diploids (Shi et al., 2021; Yang et al., 2021). The competition to achieve fertilization would be a strong selective force. Indeed, homoeologs expressed in pollen appear to have longer retention times than those with other expression patterns, which has been attributed to the stronger selection on stoichiometric change in the haploid state (Chettor et al., 2014).

## Neutral variation

Singleton genes can show significant variation in their levels of expression (Schadt et al., 2003; West et al., 2007; Zhou et al., 2020). This variation is likely to be essentially neutral, especially for nondosage-sensitive genes. While, as noted above, there are constraints on the level of expression of dosage-sensitive genes, there could also be neutral variation for the level of expression of these classes as well in certain cell types or tissues (Figure 1). Thus, variation in the level of expression of members of a duplicate pair could simply be neutral variation as occurs with singleton genes. The fact that the duplicates differ in expression is not necessarily a reflection of split or new functions. Thus, it is extremely difficult to classify the fate of duplicate genes on this basis alone.

## Intersection of fates

The fate of duplicate genes and their impact to some degree depends on the time from the duplication event. Early after duplication a certain level of buffering is operative in that deletion or mutation of one member of a pair could have little impact on the organism compared to deletion or mutation of a singleton copy. Hypofunctionalization could be a major aspect somewhat later after the initial event as the two members deteriorate to some degree. Genome dominance in which one genome in an allopolyploid is expressed at a generalized higher level than the other can have an impact on the fractionation process with the lesser expressed genome losing more genes (Schnable et al., 2011; Edger et al., 2017). Dosage balance is in play immediately after duplication and can operate over millions of years but will likely decay with time depending on the degree of connectivity. Maintenance by dosage balance of transcription factors and signaling components for longer periods of evolutionary time than other classes of genes would provide a longer period of time for new functions and split functions to arise in the very classes of genes that would generate morphological innovation. It is difficult to determine the course of neo and subfunctionalization, which potentially could arise quickly after duplication, or after long periods during which the duplicate pair is maintained by other means, but those duplicates with very extended lives likely involve these two paths.

## Funding

Research supported by National Science Foundation grant IOS-1545780.

*Conflict of interest statement.* None declared.

## Notes

The author responsible for distribution of materials integral to the findings presented in this article in accordance with the policy described in the Instructions for Authors (https://academic.oup.com/plcell) is: James A. Birchler (birchlerj@missouri.edu).
